# -2548G>A *LEP* Polymorphism Is Positively Associated with Increased Leptin and Glucose Levels in Obese Saudi Patients Irrespective of Blood Pressure Status

**DOI:** 10.3390/medicina58030346

**Published:** 2022-02-24

**Authors:** Essa M. Sabi, Lotfi S. Bin Dahman, Abdul Khader Mohammed, Khalid M. Sumaily, Nasser M. Al-Daghri

**Affiliations:** 1 Clinical Biochemistry Unit, Pathology Department, College of Medicine, King Saud University, Riyadh 11461, Saudi Arabia; ksumaily@ksu.edu.sa; 2 Chair for Biomarkers of Chronic Diseases, Department of Biochemistry, College of Science, King Saud University, Riyadh 11451, Saudi Arabia; lotfydahman@hu.edu.ye (L.S.B.D.); makhaderonline@gmail.com (A.K.M.); aldaghri2011@gmail.com (N.M.A.-D.); 3 Department of Medical Biochemistry, College of Medicine and Health Sciences, Hadhramout University, Mukalla 50511, Yemen; 4 Sharjah Institute for Medical Research, University of Sharjah, Sharjah 27272, United Arab Emirates

**Keywords:** leptin, leptin gene polymorphism, obesity, hypertension, type 2 diabetes, Saudi Arabia

## Abstract

*Background and Objectives*: In this study, we aimed to investigate the link between common -2548G>A (rs7799039) promoter variant of the human leptin gene (*LEP*) with leptin and serum glucose leptin levels in obese Saudi patients. *Materials and Methods*: A total of 206 Saudi adults (80 obese normotensive nondiabetics, 76 obese hypertensive with Type 2 Diabetes and 50 normotensive nondiabetic controls) were genotyped for -2548G>A *LEP* polymorphism using the polymerase chain reaction-restriction fragment-length polymorphism technique. *Results:* Participants with minor AA genotype had significantly higher blood glucose levels (6.8 ± 0.55 mmol/L vs. 5.8 ± 0.30 mmol/L; *p* < 0.04) and HOMA-IR (4.1 ± 0.84 vs. 2.6 ± 0.67; *p* = 0.03) against those carrying major GG genotype. Participants with heterozygous GA genotype had significantly higher serum leptin levels against those carrying major GG genotype (40.0 ± 2.6 ng/mL vs. 29.6 ± 2.6 ng/mL; *p* = 0.04). Further investigation showed that individuals with AA, GA, GA + AA genotypes are at greater risk of developing hyperglycemia compared to those with GG genotype [OR 3.7(1.6–8.4), *p* = 0.001; 3.2 (1.2–8.6), *p* = 0.03; 3.5 (1.6–7.7), *p* = 0.001, respectively]. Additionally, the -2548AA allele was shown to be a risk factor for hyperglycemia [OR 1.9 (1.2–3.0), *p* = 0.006]. Our data revealed no relationship between this variant of the LEP gene with systolic and diastolic BP, signifying that this genetic variant is not a significant marker of obesity and hypertension in the Saudi population. *Conclusions*: AA and GA genotypes and *LEP* gene -2548AA alleles may signify potent risk factors predisposing healthy individuals to develop T2DM regardless of blood-pressure profile.

## 1. Introduction

Obesity is associated with diabetes mellitus (T2DM), cardiovascular disease and hypertension, and is one of the most serious health problems of the last century [1,2]. Most patients with hypertension suffer from an increased body mass index (BMI) [3], along with 7.6 million early deaths being attributed to hypertension [4]. The prevalence of obesity and hypertension in Saudi Arabia is 35% and 30.2%, correspondingly [5,6]. Even though the link between blood pressure and increased body mass has been reported [7,8] these associations are not entirely understood [9].

Leptin (16-kDa protein) is comprised of 164 amino acids and is predominantly produced by white adipose tissue. Leptin plays an important role in regulating body weight by preventing food intake and stimulating energy expenditure [10]. Furthermore, leptin appears to play a role in the development of hypertension. As a result, leptin-mediated sympathetic activation carried in the circulatory system and at the renal stage could potentially impact BP management and lead to obesity-related hypertension [11,12].

The leptin (*LEP*) gene located at chromosomal location 7q31.3 includes three introns and three exons, spanning approximately 18 kb [13,14]. A common variant of the -2548G>A (rs7799039) leptin promoter results in the transition substitution of guanine to adenine at nucleotide position -2548 upstream of the ATG start site [15]. This variant has previously been reported to be association with elevated serum leptin levels, metabolic syndrome, hypertension and obesity [16,17,18,19,20,21,22]. On the other hand, there is conflicting information relating to these associations with some studies reporting a positive correlation and some reporting a negative correlation. Therefore, this study aimed to investigate the link between the -2548G>A *LEP* polymorphism with elevated serum glucose and leptin levels in obese Saudi patients.

## 2. Subjects and Methods

### 2.1. Subjects

A total of 206 Saudi adults aged ≥35 years old (112 females and 94 males) were randomly recruited from seventeen thousand consenting Saudi subjects in the RIYADH COHORT -Biomarker Screening in Riyadh Project. The study subjects were subdivided into 80 obese normotensive nondiabetics (ND), 50 normotensive nondiabetic (ND) controls, and 76 obese hypertensives with T2DM. Patients were informed of the study protocol, and were asked for their informed consent. To scrutinize the genetic cause of the patients, the exclusion criteria considered were as follows: regular use of antihypertensive drugs, chronic liver disease, kidney disease, cardiovascular problems, cancers and patients with acute comorbidities that required immediate medical attentions. The Declaration of Helsinki was followed and the present study was approved by the ethical committee at King Saud Medical City, Ministry of Health, Kingdom of Saudi Arabia on 17-1-2012 with reference number 11395.

### 2.2. Anthropometric and Blood Pressure Measurements

According to the World health organization (WHO) standard, anthropometric measurements obtained from all participants include weight (kg), height (cm), waist (cm), hips (cm), and hip circumference ratio (WHR) [23]. The Holtain Khan abdominal caliper from Holtain Ltd. (Crymych, UK) measured the sagittal abdominal diameter as previously reported by Al-Daghri et al. [24]. The standard equation of weight in kilogram/height in centimeter (kg/m^2^) was used to calculate body mass index (BMI). Obese subjects were defined as BMI ≥ 30 kg/m^2^ and normal weight subjects were identified via a BMI of 18–25, according to WHO guidelines. Patients with systolic blood pressure ≥140 mmHg and diastolic blood pressure ≥90 mmHg were defined as having hypertension [25]. Diabetic patients were diagnosed based on medical history, regular anti-diabetic medications, or the American Diabetes Association (ADA) criteria [26]. Patients with T2DM were defined as fasting blood glucose level ≥ 7.1 mmol/L, 2 h postprandial plasma glucose level ≥ 11.1 mmol/L) [26].

### 2.3. Blood Sampling and Biochemical Analyses

Whole blood (10 mL) was collected from all participants, after overnight fasting, by vein puncture in tubes with EDTA for genomic DNA extraction. The collected blood samples were immediately transported to the laboratory. Separation of serum was performed and was stored at −20 °C for biochemical analyses. A chemical analyzer was employed to determine blood glucose, triglycerides, total cholesterol and High density lipoprotein (HDL) cholesterol (Konelab, Finland). Friedwald’s formula was used to measure Low density lipoprotein (LDL) cholesterol concentrations [27]. Serum leptin and insulin levels were analyzed using Luminex Milliplex kits (Luminexcorp, Austin, TX, USA). Insulin sensitivity was assessed by the homeostasis model assessment of insulin resistance (HOMA-IR), calculated using the following formulae: fasting insulin (IU/mL) X fasting glucose (mmol/L)/22.5 [28]. All biochemical assays were carried out using duplicates and the average obtained from two values was considered for data analyses. Duplicate samples that failed to provide a coefficient of variation of <15% were re-analyzed. The intra-assay and inter-assay coefficients of variation (CV) for serum leptin was (7.9% and 15%) and insulin (5.1% and 14%), respectively.

### 2.4. Genetic Analyses of LEP G-2548A Polymorphism

Blood DNA was extraction was performed using a Blood genomic mini spin kit (GE Health Care, UK). At the same time, the concentration and purity of the DNA were determined using the Nanodrop spectrophotometer. The -2548G>A *LEP* polymorphism (rs7799039) was analyzed using Polymerase chain reaction-restriction enzyme length polymorphism (PCR-RFLP). A PCR mix (Taq ready mix, Kapa Biosystems, Wilmington, MA, USA) and the following primers: 5′AAAGCAAAGACAGGCATA AAAA-3′ (antisense) and 5′-TTTCCTGTAATTTTC CCGTGAG-3′ (sense), were employed for the amplification of the 242bp DNA fragment. Conditions for PCR were set as mentioned by Mammés et al. [10]. The PCR reaction in the final reaction volume of 50 µL contained genomic DNA (50 ng), 0.4 µM of primer (sense and antisense) and KAPA Taq ready mix (25 µL) DNA polymerase. PCR condition was as following: initial denaturation of 5 min at 95 °C followed by 35 cycles with each cycle containing denaturation at for 15 s at 94 °C, 30 s at 52 °C (annealing), 30 s at 72 °C (extension), and followed by final extension for 5 min at 72 °C. The amplified DNA products were digested for 2 h at 37 °C with 2 U of *HhaI* restriction enzyme further separated on 2.5% agarose gels with stain (ethidium bromide). The 242 bp PCR product (AA) was digested into 181 bp (GG) and 61 bp (GA) fragments in the presence of a G nucleotide (polymorphic variant) but not in its absence.

### 2.5. Statistical Analysis

The mean and standard deviation (SD) were used to express the data. The parameters of the analyzed groups were compared using an analysis of covariance (ANCOVA). Analysis of covariance (ANCOVA) was performed, adjusting for the potential confounder of age. Odds ratios (OR) and 95% confidence intervals were measured by logistic regression (multinomial) for the allele and genotype frequencies of -2548G>A *LEP* polymorphism. Data were analyzed using the Statistical Package for the Social Sciences for Windows (SPSS ver16.0). *A p*-value of <0.05 was considered statistically significant.

## 3. Results

### 3.1. Anthropometric and Biochemical Characteristics of Participants

Biochemical and anthropometric characteristics of the participants are represented in Table 1. There was no significant difference for the mean age of the study groups (*p* = 0.14) as shown in Table 1. Obese hypertensive with T2D and obese normotensive ND patients had significantly higher systolic BP (*p* = 0.01, *p* < 0.001), diastolic BP (*p* = 0.002, *p* < 0.001), BMI (*p* < 0.001, *p* < 0.001), waist (*p* < 0.001, *p* < 0.001), hips (*p* = 0.001, *p* < 0.001) and SAD (*p* < 0.001, *p* < 0.001), respectively matched with normotensive ND controls. Obese hypertensive with T2D patients had significantly higher levels of HDL-cholesterol (*p* = 0.04) than the obese normotensive ND. Obese hypertensive with T2D patients had significantly higher serum glucose levels (*p* = 0.009) than normotensive ND controls and obese normotensive ND. Further, Obese normotensive ND and obese hypertensive with T2D patients had elevated serum leptin levels (*p* < 0.001, *p* < 0.001) and HOMA-IR (*p* = 0.01) when compared with normotensive ND controls, while no significant difference in serum total cholesterol (*p* = 0.60), LDL-cholesterol (*p* = 0.99), triglycerides (*p* = 0.08), and insulin (*p* = 0.15) was observed.

### 3.2. Distribution of Alleles and Genotype Frequency for -2548G>A LEP Polymorphism

PCR RFLP analysis of the -2548G>A *LEP* provided three different variants of the genotype, namely GA heterozygous, GG for the wild type and AA for the homozygous (mutant type). For controls-normotensive ND, the genotype frequencies of the -2548G>A *LEP* polymorphism were 40.0% (GA), 46.0% (GG) and 14.0% (AA). With regard to obese normotensive ND, individuals with GA, GG, and AA genotypes were 38.8%, 40.0%, and 21.2%, respectively. Furthermore, obese hypertensive T2D patients had GA, GG and AA frequencies of 48.7%, 33.0%, and 18.3%, respectively (Table 2). Furthermore, the -2548GG allele was 66% in normotensive ND controls. Additionally, 59.4% of the -2548G allele was found in obese normotensive ND and 57.2% in obese hypertensive with T2D patients. The -2548AA allele frequencies were 34.0% in normotensive ND controls, obese normotensive ND patients had 40.6%, and obese hypertensive T2D patients had 42.8% (Table 2). Additionally, the relationship between metabolic disorders (BMI, diabetes, and hypertension) and the -2548G>A *LEP* polymorphism is presented in Table 2. We found no significant difference in allele or genotype frequencies between obese hypertensive T2D compared to normotensive ND controls.

### 3.3. Anthropometric and Clinical Characteristics of the Study Population

The characteristics of the anthropometric and biochemical parameters with respect to the -2548G>A LEP polymorphism are presented in Table 3. There was no significant association between the genotypes of the -2548G>A LEP polymorphism with systolic BP, diastolic BP, BMI, hips and waist. In the study group, AA genotype carriers were significantly associated with increased blood glucose levels (*p* < 0.04) and HOMA-IR (*p* = 0.03). In contrast, the GA genotype was significantly associated with increased serum leptin levels (*p* = 0.04). Additionally, in normotensive ND controls, the GA genotype was significantly associated with increased leptin levels (*p* = 0.04). In contrast, no significant association between the genotypes of this gene and serum total cholesterol, HDL-cholesterol, LDL-cholesterol, triglyceride, and insulin were observed (Table 3).

**Figure 1:**
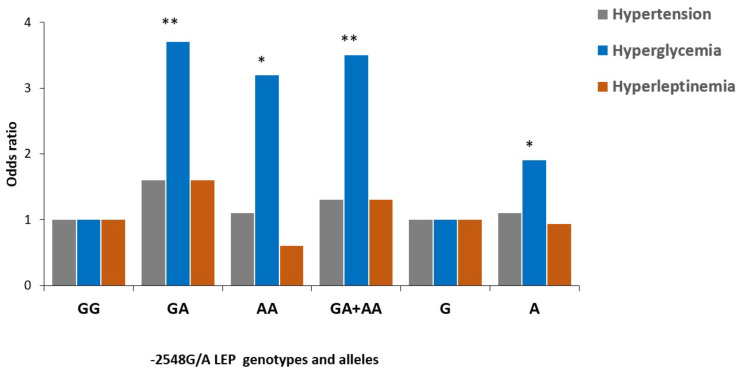
Genotypes and alleles distribution of the -2548G/A *LEP* polymorphism in individuals with normal vs. elevated blood pressure (blood pressure ≥ 140/90) and normal vs. elevated blood glucose (≥7.0 mmol/L) participants; as well as normal vs. hyperleptinemia Subjects (9 ng/mL males and 27 ng/mL). GG is considered as a reference genotype. G allele is considered as a reference allele * denotes *p* < 0.05, ** denotes *p* < 0.005.

Further analysis showed that the individuals carrying GA, AA, GA + AA genotypes were more susceptible to the development of hyperglycemia matched to those carrying the GG genotype [OR 3.7 (1.6–8.4), *p* = 0.001; 3.2(1.2–8.6), *p* = 0.03; 3.5 (1.6–7.7), *p* = 0.001, respectively] (Figure 1). Additionally, the -2548A A allele was found to be a risk factor of diabetes in individuals with hyperglycemia [OR 1.9 (1.2–3.0), *p* = 0.006] (Figure 1). However, no such association was found between hypertension and hyperleptinemia.

## 4. Discussion

Previously, *LEP* was sequenced and analyzed for variants that are potentially associated with the pathophysiology of obesity and obesity-related complications such as T2DM and hypertension. One of these common polymorphisms is -2548G>A with studies reported polymorphism to be associated with BMI among overweight Europeans [10], Tunisian volunteers [29]. Additionally, other studies show the link between obesity and the variant of the -2548G>A *LEP* [19,30,31]. However, there is conflicting evidence amongst different ethnicities for associations with BMI and obesity. Wang et al. found that individuals with a higher BMI had a significantly higher GG (wild-type, reference sequence) genotype than those with GA or AA genotypes in the Taiwanese aboriginal community [20]. Similarly, researchers from Tunisia [18], Turkey [32], and Romania [33] reported no association between the -2548G>A *LEP* polymorphism and higher BMI. Furthermore, other studies also have described no association between the -2548G>A *LEP* polymorphism and obesity in Serbian [34], Spanish [35], Romanian [36], and Turkish obese patients [37].

In our study, there was a significant difference in the allelic distribution of patients compared with the healthy controls, having a probable effect on the risk of diabetes. The present study showed no association between this variant of the *LEP* gene with BMI, WHR, SAD, systolic and diastolic BP, indicating that this genetic variant is not a significant marker of hypertension and obesity in the Saudi population. Thus, we suggest that other potential contributing factors such as lifestyle, physical activity, ethnicity, nutritional and environmental factors may affect energy homeostasis. Thus, the GA and AA genotype carriers may present a chance to develop hyperglycemia compared to those with the GG genotype. Additionally, the -2548A allele was identified as a risk factor of diabetes in individuals with hyperglycemia against those carrying the -2548G allele. The study also indicates that the GA genotype carriers had a significant association with increased leptin and serum levels.

Since the variant of -2548G>A *LEP* is located at the 5’ end in the promoter region, Gong et al. suggested this variant may effect *LEP* gene expression and secretion via adipose tissue [38]. Moreover, the -2548G>A *LEP* polymorphism is close to the Sp1 transcription-factor binding site and two repetitive sequences of MER11 and Alu, regulating LEP transcription [39]. Furthermore, another study reported that the Sp1 binding site has a potent role in the transcriptional activation of the LEP promoter by insulin-mediated glucose metabolism [40]. However, the potential effects of the -2548G>A *LEP* polymorphism on leptin secretion and expression are still conflicting. For example, Tunisian obese individuals with the -2548AA allele had lower plasma leptin levels than those carrying the -2548GG allele [18]. On the other hand, Yiannakouris et al. [41] reported a significant association between the -2548GG allele and elevated serum leptin levels in Greek healthy individuals.

Additionally, the relationship among the -2548G variant and increased serum leptin levels was reported in European [34], Brazilian [19], and Romanian obese individuals [25]. The AA genotype of -2548G>A LEP, on the other hand, was linked to higher serum leptin levels in the French men cohort study [10]. A Swedish study confirmed this finding, reporting that the -2548A allele was linked with elevated messenger RNA (mRNA) levels and adipose tissue leptin secretion rate [17]. In our study findings, only obese individuals with the GA genotype had increased leptin serum levels compared to those with the GG genotype. Based on the above findings, these conflicting results could arise from the presence of the -2548G>A *LEP* polymorphism and other polymorphisms in leptin and leptin gene receptors, which were not assessed as part of this study. As previously identified in other studies showing different results from diverse backgrounds, the ethnicity and characteristics of Saudi individuals could also account for the conflicting findings. Additionally, sample size is a limitation of this study, and this may account for the conflicting results identified in this study. In addition, the study described herein did not measure HbA1c and adiponectin as metabolic markers of obesity and obesity-related complications.

Leptin suppresses glucose-stimulated insulin secretion and receptors of leptin on β cells and fat cells, allowing it to control insulin secretion and action, or both [42]. Our study found that the AA genotype of -2548G>A *LEP* polymorphism was significantly associated with elevated blood glucose levels and HOMA-IR. Our results agree with data from Vaškù et al. [43], who suggested that the AA and AG genotype carriers have a significantly higher risk for gestational diabetes mellitus than those carrying the GG genotype. Additionally, a recent study in the North Indian population identified the rs7799039 AA genotype as a risk factor for T2DM [44]. However, these findings contradict those of a recent study, which found that carriers of the -2548GG allele were linked to higher blood glucose levels in T2DM patients [45]. Thus, based on our data, we suggest that the GA and AA genotype carriers might be a risk factor for T2DM as opposed to those carrying the GG genotype, which is in agreement with the hypothesis that leptin has a potent role in the development of insulin resistance among T2DM patients.

The role of leptin in the obesity-related hypertension pathogenesis appears to be a result of the circulatory system, renal activity & modulation and sympathetic nervous system (SNS) activation [12,14] and can be prevented by adrenergic blockade drugs [11]. Elevated renal sympathetic nerve activity, which results in salt retention, volume expansion, and increased blood pressure, is one of the main outcomes of SNS activation in hypertension condition [44]. In addition to SNS activation, leptin induces the expression of the endothelin type A receptor in vascular smooth muscle cells and activates endothelin-1 production in vascular endothelial cells [46], and smooth muscle cell proliferation [47]. Previous study groups identified a positive association between systolic and diastolic blood pressure with the -2548G>A *LEP* polymorphism. Among Tunisian obese (males), Ben Ali et al. [48] found that individuals with higher AA genotype have increased systolic and diastolic blood pressure against those carrying GG genotype. Brazilian obese patients have shown that the AA genotype had substantially lower systolic, diastolic, and mean arterial blood pressure [49]. However, our study showed no association between the -2548G>A *LEP* polymorphism and hypertension. This difference in the relation between the -2548G>A *LEP* polymorphism and blood pressure might be attributable to the difference in serum estrogen concentration between men and women, given that estrogen exerts various effects on the stimulation of nitric oxide production and prostaglandin as well as the inhibition of endothelin-1 release by vascular endothelial cells [50]. Hu et al. [51] reported that the relationship between increased serum leptin levels and blood pressure was heavily influenced by body-fat mass and distribution in a rural Chinese population. Another reason for this relation is hyperinsulinemia and insulin resistance and their association with hypertension. El-Gharbawy et al. [52] found a strong association between increased serum leptin levels and blood pressure in obese African women with hypertension, which disappeared after adjustment for another component of the insulin resistance syndrome.

One limitation of the present study was the smaller sample size. Another limitation is that we did not measure HbA1c and adiponectin as metabolic markers of obesity and obesity-related complications. Finally, this study also did not analyze leptin gene receptors together with the -2548G> A LEP polymorphism.

In this study, the differences in significance observed in terms of glycaemia, HOMA-IR and leptinemia and their association with the studied genetic variant was due to the size of sample used and gender (male female ratio). This is the reason that we observed significant associations when we consider the population overall but no significance was observed when they were sub grouped.

## 5. Conclusions

The current study found that the genotype distribution of the -2548G> A variant of *LEP* was associated with serum glucose and leptin levels in a set of Saudi obese individuals. Additionally, AA and GA genotypes and the -2548AA allele could be a potential risk factors for predisposing healthy subjects to T2DM. However, an extensive study with a larger sample size should be used to identify the real implication of correlations between the LEP polymorphism and diabetes mellitus, strengthening evidence for the LEP polymorphism as a genetic factor in diabetes mellitus risk.

## Figures and Tables

**Table 1:** Anthropometric and clinical characteristics of the study population.

Parameter	Normotensive ND Control	Obese Normotensive Nondiabetics (ND)	Obese Hypertensives with T2D	*p*-Value
N	50	80	76	
Age	47.9 ± 5.4	47.7 ± 6.0	49.4 ± 5.9	0.14
Gender (M\F)	(32/18)	(37/43)	(25/51)	0.003
BMI (kg/m^2^)	22.9 ± 2.1	34.1 ± 4.2 *	35.1 ± 4.7 *	<0.001
Waist (cm)	84.7 ± 14.6	99.1 ± 16.6 *	99.1 ± 20.3 *	<0.001
Hips (cm)	96.5 ± 17.1	110.4 ± 19.6 *	111.3 ± 24.0 *	<0.001
SAD (cm)	19.6 ± 4.0	24.9 ± 5.5 *	25.5 ± 4.1*	<0.001
Systolic BP (mmHg)	116.7 ± 8.6	121.9 ± 10.4 *	144.1 ± 11.2 *^,**†**^	<0.001
Diastolic BP (mmHg)	75.3 ± 5.6	78.8 ± 6.2 *	91.2 ± 4.0 *^,**†**^	<0.001
Leptin (ng/mL)	15.6 ± 5.2	38.8 ± 6.2 *	38.6 ± 5.1 *	<0.001
HDL (mmol/L)	0.83 ± 0.30	0.74 ± 0.31	0.86 ± 0.32 ^**†**^	0.04
TC (mmol/L)	5.3 ± 1.1	5.4 ± 1.2	5.5 ± 1.1	0.59
LDL (mmol/L)	3.8 ± 1.0	3.8 ± 1.1	3.8 ± 1.0	0.99
TG (mmol/L)	1.5 ± 0.24	1.8 ± 0.39	1.8 ± 0.35	0.08
FBG (mmol/L)	5.8 ± 2.0	6.4 ± 2.3	7.2 ± 3.1 *	0.01
Insulin (IU/mL)	9.4 ± 2.5	11.4 ± 1.2	12.3 ± 1.2	0.15
HOMA-IR	2.4 ± 0.85	3.1 ± 0.64 *	3.8 ± 0.72 *	0.01

Level of significance at *p* < 0.05; * represents group 2 and 3 is significantly different normotensive ND control, **†** represents group 3 is significantly different obese normotensive ND. Analysis of covariance (ANCOVA) is done; age was set as a covariance. ND: non-diabetes; T2D: type two diabetes; SAD: sagittal abdominal diameter; BMI: body mass index; diastolic BP: diastolic blood pressure; systolic BP: systolic blood pressure; TC: total cholesterol; HDL: high-density lipoprotein; LDL: low-density lipoprotein; TG: triglyceride; HOMA-IR: homeostasis model assessment of insulin resistance; FBG: fasting blood glucose. All data is represented by mean ± standard deviation (SD).

**Table 2:** Genotype and alleles frequency distribution of the -2548G/A *LEP* polymorphism.

	Normotensive	Obese Normotensive			Obese Hypertensive		
	ND Control	ND			T2D		
	N (in %)		Odds ratio (95%CI)	*p*-value	N (in %)	Odds ratio (95%CI)	*p*-value
Genotype frequency							
GG	23 (46.0)	32 (40.0)	1		25 (33.0)	1	
GA	20 (40.0)	31 (38.8)	1.1 (0.51–2.4)	0.84	37 (48.7)	1.7 (0.77–3.7)	0.23
AA	7 (14.0)	17 (21.2)	1.7 (0.62–4.8)	0.32	14 (18.3)	1.8 (0.63–5.3)	0.30
GA + AA	27 (64.0)	48 (60.0)	1.3 (0.62–2.6)	0.58	51 (67.0)	1.0 (0.49–2.1)	0.90
Allele frequency							
G allele	66 (66.0)	95 (59.4)	1		87 (57.2)	1	
A allele	34 (34.0)	65 (40.6)	1.3 (0.78–2.2)	0.29	65 (42.8)	1.4 (0.85–2.4)	0.18

Data are represented as n (%). Significant difference from homozygous GG. ND: non-diabetic; T2D: type two diabetes. GG, GA and AA are genotypes.

**Table 3:** Anthropometric and clinical characteristics of the study population with respect to the -2548G/A *LEP* polymorphism.

	Overall			Normotensive ND Healthy Control Group			Obese Normotensive ND Group			Obese Hypertensive T2D Group		
	GG	GA	AA	GG	GA	AA	GG	GA	AA	GG	GA	AA
N	80	88	38	23	20	7	32	31	17	25	37	14
Age	48.4 ± 6.0	48.6 ± 5.8	47.9 ± 5.6	48.2 ± 5.7	46.9 ± 5.2	49.8 ± 5.3	48.5 ± 5.7	47.4 ± 6.1	46.7 ± 6.4	48.5 ± 7.0	50.4 ± 5.6	48.4 ± 4.8
Gender (M/F)	(39/41)	(34/54)	(21/17)	(15/8)	(12/8)	(5/2)	(17/15)	(10/21)	(10/7)	(7/18)	(12/25)	(6/8)
BMI (kg/m^2^)	31.6 ± 6.7	32.2 ± 6.4	31.5 ± 6.1	23.2 ± 2.6	23.0 ± 1.2	21.7 ± 1.4	33.9 ± 5.1	34.9 ± 4.0	32.6 ± 2.5	35.6 ± 4.1	34.8 ± 4.8	35.1 ± 5.3
Waist (cm)	97.1 ± 17.7	94.9 ± 18.8	93.3 ± 20.4	87.5 ± 9.6	83.8 ± 10.8	78.1 ± 11.6	100.1 ± 16.3	97.5 ± 19.8	100.4 ± 10.2	102.2 ± 21.9	98.9 ± 19.4	94.1 ± 20.2
Hips (cm)	109.2 ± 20.3	106.6 ± 22.5	104.7 ± 23.1	97.8 ± 9.3	100.1 ± 16.2	80.1 ± 11.0	110.4 ± 19.6	109.1 ± 22.8	100.1 ± 10.2	118.1 ± 24.0	107.9 ± 24.6	107.5 ± 21.8
SAD (cm)	23.3 ± 4.7	24.6 ± 5.4	22.6 ± 2.7	19.9 ± 3.9	19.8 ± 4.2	18.4 ± 4.4	23.8 ± 5.0	26.3 ± 5.3	23.4 ± 7.1	26.1 ± 2.8	25.6 ± 4.6	24.2 ± 4.5
Systolic BP (mmHg)	127.5 ± 16.1	130.2 ± 16.8	128.4 ± 12.1	114.8 ± 8.4	117.5 ± 9.0	120.7 ± 7.3	121.8 ± 9.8	122.3 ± 12.5	121.5 ± 7.4	146.5 ± 8.9	143.8 ± 13.3	140.7 ± 8.0
Diastolic BP (mmHg)	81.7 ± 8.6	83.1 ± 9.4	82.7 ± 6.6	74.9 ± 8.5	75.0 ± 6.1	77.1 ± 3.9	78.9 ± 6.1	78.4 ± 6.9	79.4 ± 5.2	91.6 ± 3.4	91.6 ± 5.0	89.6 ± 2.0
TC (mmol/L)	5.4 ± 1.3	5.5 ± 1.0	5.3 ± 1.1	5.3 ± 1.2	5.2 ± 0.90	5.9 ± 1.2	5.6 ± 1.4	5.4 ± 1.0	5.1 ± 1.0	5.3 ± 1.2	5.8 ± 1.1	5.4 ± 1.0
TG (mmol/L)	1.82 ± 0.10	1.85 ± 0.11	1.86 ± 0.21	1.6 ± 0.17	1.4 ± 0.10	1.5 ± 0.26	2.0 ± 1.0	1.8 ± 1.1	2.1 ± 1.8	1.8 ± 0.20	2.1 ± 0.20	1.7 ± 0.15
HDL (mmol/L)	0.75 ± 0.17	0.81 ± 0.17	0.74 ± 0.22	0.75 ± 0.18	0.86 ± 0.13	0.80 ± 0.24	0.73 ± 0.17	0.75 ± 0.18	0.57 ± 0.17	0.76 ± 0.16	0.83 ± 0.17	0.96 ± 0.20
LDL (mmol/L)	3.8 ± 1.0	3.8 ± 0.99	3.7 ± 0.99	3.7 ± 1.1	3.6 ± 0.75	4.3 ± 0.81	3.9 ± 1.2	3.7 ± 1.0	3.5 ± 1.1	3.6 ± 0.20	4.0 ± 1.0	3.5 ± 1.0
Leptin (ng/mL)	29.6 ± 2.6	40.0 ± 2.6 *	26.8 ± 2.3	12.3 ± 1.8	26.1 ± 4.2 *	23.2 ± 3.5	38.8 ± 2.7	47.7 ± 2.2	24.4 ± 2.1	38.3 ± 2.4	41.6 ± 1.8	31.5 ± 2.0
FBG (mmol/L)	5.8 ± 0.30	6.6 ± 0.50	6.8 ± 0.55 *	5.8 ± 0.16	5.1 ± 0.20	7.1 ± 0.76	5.6 ± 0.29	6.8 ± 0.48	6.3 ± 0.38	6.1 ± 0.37	7.3 ± 0.55	7.6 ± 0.61
Insulin (IU/mL)	10.2 ± 1.2	11.2 ± 1.3	13.0 ± 1.2	8.2 ± 1.8	9.4 ± 1.6	13.2 ± 1.7	11.0 ± 1.1	10.6 ± 1.1	13.0 ± 1.2	11.1 ± 1.0	12.8 ± 1.1	12.8 ± 1.0
HOMA-IR	2.6 ± 0.67	3.3 ± 0.73	4.1 ± 0.84 *	2.2 ± 0.88	2.2 ± 0.80	3.8 ± 0.88	2.7 ± 0.54	3.2 ± 0.68	3.7 ± 0.75	3.0 ± 0.62	4.1 ± 0.68	4.5 ± 0.95

Data represented by mean ± standard deviation. Level of significance at *p* < 0.05; * represents group is significantly different from Homozygous GG. ND: non-diabetes; T2D: type two diabetes; SAD: sagittal abdominal diameter; diastolic BP: diastolic blood pressure; systolic BP: systolic blood pressure; TC: total cholesterol; TG: triglyceride; HDL: high-density lipoprotein; LDL: low -density lipoprotein; FBG: fasting blood glucose; HOMA-IR: homeostasis model assessment of insulin resistance and BMI: body mass index.

## Data Availability

The data that supports the findings of this study are all available in the manuscript.
